# Nurses' Sleep Quality and Its Influencing Factors During the First Explosive COVID‐19 Outbreak in Zhejiang, China, After the Relaxation of Epidemic Prevention and Control Measures: A Multicentre Cross‐Sectional Study

**DOI:** 10.1002/nop2.70127

**Published:** 2025-01-23

**Authors:** Lifen Lu, Di Sheng, Yaling Zhu, Xiaowei Xia, Guanghui Chen, Jiali Liang, Xiulan Shen, Gui Zheng

**Affiliations:** ^1^ Department of Nursing, the First Affiliated Hospital Zhejiang University School of Medicine Hangzhou Zhejiang P.R. China; ^2^ Department of Nursing The First Hospital of Jiaxing Jiaxing Zhejiang P.R. China; ^3^ Ministry of Health The Third Hospital of Ninghai County Ningbo Zhejiang P.R. China; ^4^ Ministry of Health The Liangzhu Street Community Health Service Center Hangzhou Zhejiang P.R. China; ^5^ Department of Nursing The First Affiliated Hospital Zhejiang University School of Medicine Hangzhou Zhejiang P.R. China

**Keywords:** COVID‐19 outbreak, influencing factor, nurses, sleep quality

## Abstract

**Aim:**

To investigate the sleep quality and its influencing factors among nurses in hospitals in Zhejiang, China, during the first explosive COVID‐19 outbreak following the relaxation of prevention and control measures.

**Design:**

A multicentre cross‐sectional study.

**Methods:**

Between 10 January and 20 January 2023—approximately 1 month after the policy was loosened—a total of 573 nurses from tertiary and community hospitals in Zhejiang participated in an online, self‐administered survey. The participants were recruited using convenience sampling, and the survey was distributed via the WeChat platform. The survey included a general information form; the Self‐Rating Scale of Sleep (SRSS); the Depression, Anxiety, and Stress Scale‐21 (DASS‐21); the Perceived Social Support Scale (PSSS); and the Brief Resilience Scale (BRS). Data analysis was conducted using SPSS version 26.0. Statistical methods employed included *t*‐tests, one‐way analysis of variance (ANOVA), chi‐square tests and Mann–Whitney *U*‐tests for comparisons between groups. Pearson correlation coefficients were calculated to analyse the relationship between the SRSS score and the DASS‐21, PSSS and BRS scores. A multiple linear stepwise regression analysis was conducted to determine the independent influencing factors of sleep quality.

**Results:**

More than 90% of the nurses were infected with COVID‐19, and 60.6% had sleep disorders. The regression analysis revealed that anxiety, the BRS score, comorbidities, hospital grade, clinical front‐line, age and COVID‐19 infection independently predicted sleep quality. The scores for several SRSS items were higher than the Chinese norm, especially for the nurses in tertiary hospitals.

**Patient and Public Contribution:**

No patient or public contribution.

## 1 Introduction

On 11 November 2022, the Chinese government issued the Notice on Further Optimising the Prevention and Control Measures Against the Novel Coronavirus (COVID‐19) and Ensuring Scientific and Accurate Prevention and Control (https://www.gov.cn/xinwen/2022‐11/11/conten_5726122.htm). This was followed by announcements from Chinese provincial governments. For instance, several regions in Zhejiang Province issued the Notice on Optimising and Adjusting Relevant Measures for Epidemic Prevention and Control (https://zjnews.zjol.com.cn/zjnews/202212/t20221204_25144742.shtml) on 4 December. This indicated that China was preparing to end its 3‐year‐long approach of strict prevention and resolute defence against the epidemic. The announcement came 48 days before the Chinese New Year. As epidemic prevention and control measures were gradually lifted in major cities in China, many people developed COVID‐19 symptoms such as high fever, severe cough and body aches. This led to an unusual situation where, within a short period, cities experienced widespread disruptions, including shops and schools being closed and factories being shut down. Similarly, a significant number of nurses working in hospitals exhibited COVID‐19 symptoms; however, hospital operations could not be halted due to their critical role in healthcare delivery. The volume of fever‐related outpatient visits increased exponentially to more than 20 times the daily average, leading to congested facilities. A large number of seriously ill patients flooded into hospitals, resulting in a shortage of emergency and intensive care unit beds. There was a critical lack of medical and human resources in fever clinics and emergency departments. The pandemic has brought numerous ethical and moral challenges to public policy. For instance, issues such as ‘overloaded medical staff’ and ‘widespread public panic and concern stemming from a lack of knowledge about the disease’ have been highlighted (Hadian et al. 2022). These challenges have significantly disrupted the healthcare environment and procedural efficiency, further exacerbating the strain on healthcare systems. To address the unprecedented epidemic, hospitals in China implemented emergency measures, including integrating bed and staff management, expanding fever outpatient and emergency care facilities, converting general wards into makeshift intensive care units and deploying medical staff across specialties. In response, the government introduced additional measures, such as providing anti‐epidemic subsidies for medical workers, establishing temporary pharmacies in communities and distributing fever‐reducing drugs through retail outlets. Although the Omicron variant became the predominant strain of COVID‐19 and its pathogenic, severe and fatality rates greatly decreased, many older people with chronic medical conditions still succumbed to the virus. Consequently, people endured a challenging process involving obtaining medicine, seeing a doctor and being sent to the hospital.

A previous meta‐analysis (Jahrami et al. 2022) revealed that the estimated prevalence of sleep difficulties among healthcare professionals (42.47% [37.95%–47.12%]) was greater than that among the general population (36.73% [32.32%–41.38%]). During the COVID‐19 pandemic, a multicentre cross‐sectional survey of front‐line clinical nurses in China revealed that 80.4% had sleep disruptions, which led to increased levels of exhaustion and mental strain (Liu et al. 2022). Factors such as age, sex, lifestyle preferences and comorbidities have been shown to affect how well individuals sleep (Shim and Kang 2017). Additionally, occupational factors play a significant role (Dong et al. 2017; Karakaş, Gönültaş, and Okanlı 2017). For instance, night‐shift nurses are more likely to have physical health problems and sleep disorders than other nurses (Feng et al. 2021). Several studies have also shown a circular causal relationship between sleep quality, stress, anxiety, depression, resilience and quality of life (Hwang and Lee 2023; Jiang et al. 2021; Kang et al. 2020; Mao et al. 2023; Olagunju et al. 2021; Zhang et al. 2021). Studies indicate that family support plays a significant role in improving sleep quality among nurses, highlighting its protective effect in stressful environments (Haseli et al. 2023; Huang et al. 2022).

Existing research using the Sleep Self‐Rating Scale (SRSS) to assess sleep quality remains limited (Jiang et al. 2021; Wang et al. 2021; Zhou et al. 2021, 2022). Most studies to date have focused on contexts such as the lockdown period at the onset of the pandemic, when infections among healthcare workers were relatively rare, and external support from nurses was available. However, reflecting on the 3 years of the pandemic, it is evident that while Chinese nurses have gained substantial experience in COVID‐19 prevention and control, their direct experience with treatment and patient care has been comparatively limited.

The current surge in COVID‐19 cases has brought unprecedented challenges. Many nurses, now facing their first infections, must continue working despite illness, adhering to management protocols unless experiencing severe symptoms such as high fever. This situation is further exacerbated by the absence of external support, as nurses in individual hospitals must manage the workload independently. Compounding these challenges, the government chose to relax epidemic prevention measures during the ‘pre‐Spring Festival’ period, allowing many nurses outside Zhejiang Province to return home for the first time in 3 years.

Given these circumstances, it is crucial to reassess the sleep quality of nurses in the context of the substantial shift from strict lockdowns to a more liberalised policy during this culturally significant period. This research aims to provide a comprehensive understanding of the mechanisms underlying changes in nurses' sleep quality in response to major shifts in COVID‐19 prevention and control policies. The findings will inform the development of more humane and effective epidemic prevention programmes for future crises, alongside targeted measures such as sleep hygiene education and psychological interventions for vulnerable groups. These insights will support government and management departments in minimising the adverse impacts of similar situations in the future.

## 2 Methods

### 2.1 Study Design and Participants

In this multicentre cross‐sectional study, nurses were recruited from four hospitals in Zhejiang Province, China, using convenience sampling (Shorten and Moorley 2014). A total of 594 hospital nurses participated, including 321 tertiary hospital nurses and 273 community hospital nurses. The inclusion criteria were as follows: Registered Nurses who were at least 18 years of age, had worked in the hospital for more than 6 months and could correctly understand the questionnaire and articulate their responses were included. The exclusion criteria were as follows: nurses who had insomnia and experienced a major traumatic event within the last 6 months (e.g., death of a loved one or divorce) were excluded. According to the sample size calculation method for analysing influencing factors (Ni, Chen, and Liu 2010), the sample size should be at least 5–10 times the total number of independent variables, and an additional 20% should be added to account for potential loss of follow‐up. A total of 56 independent variables were included in this study, so the required sample size was 336–672 nurses. Each question had a ‘must answer’ condition; otherwise, the questionnaire could not be submitted. Data from 21 nurses were rejected due to a response time of less than 120 s. Finally, 573 nurses were recruited, resulting in a qualification rate of 96.46%.

### 2.2 Measurements

The following participant information was collected: sex, age, body mass index (BMI), educational level, marital status, children, place of residence, professional title, clinical frontline, length of service, income, smoking status, drinking status, religious beliefs, comorbidities, hospital grade and COVID‐19 infection.

The Chinese version of the SRSS, developed by Professor Li et al. (2000), who is the executive director of the Chinese Mental Health Association and executive editor of the *Chinese Journal of Health Psychology*, was used to assess sleep status over the previous month. A Chinese norm in the national collaboration group was also formulated. The scale contains 10 items: insufficient sleep time, poor sleep quality, daytime sleepiness, sleep hours, difficulties in falling asleep, disrupted sleep, early awakening, dreaminess or nightmares/night terrors, medication and psychophysiologic response after insomnia. Ratings were collected using a 5‐point Likert scale (1–5) to evaluate various dimensions of sleep. For example, insufficient sleep time ‘Do you think you get enough sleep?’ (1 = too much sleep, 2 = just the right amount of sleep, 3 = Slightly less sleep, 4 = not enough sleep, 5 = seriously not enough sleep); poor sleep quality ‘Do you feel rested after sleep?’ (1 = feeling well rested, 2 = feel rested, 3 = feel a little less rested, 4 = feel not rested, 5 = feel not rested at all). The total score ranged from 10 to 50, with higher scores indicating more severe sleep issues. Based on the criteria established by Li et al. (2000), scores of 22 or below indicates regular sleep, while scores of 23 or above reflect varying degrees of sleep problems: 23–29 as mild, 30–39 as moderate and 40–50 as severe. A single‐dimensional tool widely used in China has been validated in various studies (Chen et al. 2022; Zhou et al. 2022). In this study, the Chinese version of the SRSS demonstrated strong internal consistency, with a Cronbach's alpha coefficient of 0.840.

The Depression, Anxiety, and Stress Scale‐21 (DASS‐21) was employed. Compiled by Lovibond and Lovibond (1995), this 21‐item scale measures three negative emotional experiences: depression, anxiety and stress. Gong et al. (2010) introduced the simplified Chinese version of the scale for the first time and tested a group of domestic college students, obtaining good reliability, validity and structural stability. A 4‐point scale is used for scoring (0 = completely inconsistent, 1 = partially consistent, 2 = mostly consistent and 3 = fully consistent). The score for each subscale is calculated by summing the seven individual scores and then multiplying the total by 2. The total possible score ranges from 0 to 42 points, and the higher the score, the more serious the degree of depression, anxiety or stress, reflecting a worse emotional state (McMullen et al. 2018). The DASS‐21 is widely applied in China (Bai et al. 2022; Jiang et al. 2022). In this study, the psychological state over the previous month was evaluated, with the Cronbach's alpha coefficients for the depression, anxiety and stress subscales and the overall scale being 0.862, 0.837, 0.881 and 0.946, respectively.

The Chinese version of the Perceived Social Support Scale (PSSS) was used in this study. This version was compiled by Zimet et al. (1990) and translated and revised by Jiang et al. (Huang, Jiang, and Ren 1996). The scale comprises a total of 12 questions, which are divided into three categories: support from family, friends and others. It uses a 7‐point Likert scale (1 = strongly opposed, 2 = strongly opposed, 3 = slightly opposed, 4 = neutral, 5 = slightly in agreement, 6 = strongly in agreement and 7 = completely in agreement). The scale measures the perceived strength of social support, with higher scores indicating a greater degree of support. The PSSS is widely applied in China (Jing, Lu, and Yao 2022; Zheng, Morrell, and Watts 2018). In this study, the Cronbach's alpha coefficients were 0.909 for family support, 0.932 for friend support, 0.876 for other support and 0.952 for the overall scale.

The Chinese version of the Brief Resilience Scale (BRS) was used. Smith et al. (2008) compiled the scale, and Chen et al. (2020) obtained good reliability and validity of the scale. The BRS has a total of six items and is a single‐dimensional assessment tool. It includes three items each in the positive and negative directions, which are scored on a Likert scale (1 = complete disagreement, 2 = disagreement, 3 = uncertainty, 4 = agreement and 5 = full agreement). The scale gauges a person's capacity to recover health or well‐being after experiencing stress, particularly that caused by health issues or other stressful circumstances. It is the only single‐dimensional scale that assesses resilience in and of itself. The BRS is widely applied in China (Son et al. 2022). In this study, the Chinese version of the scale had a Cronbach's alpha coefficient of 0.780.

### 2.3 Data Collection

An electronic survey was created using a survey tool provided by the Chinese mainland (https://www.wjx.cn). A downloadable QR code was generated after the e‐questionnaire was edited. Hospital management distributed this QR code to department management staff (head nurses) through WeChat, one of the most widely used social media platforms in China. Head nurses then forwarded the QR code to staff nurses, who were screened based on the inclusion and exclusion criteria for this study. Data were collected from 10 January to 20 January 2023. Each IP address could be used only once to access and complete the survey. Before participating in the survey, participants provided informed consent on the first page of the questionnaire, which they accessed by scanning the WeChat code. Participants were informed of the purpose and significance of the survey, including that it was anonymous, that there was no incentive, that participation was voluntary, that participation would not affect their work and that they could withdraw at any time. Participants could only proceed with the survey by clicking the ‘agree’ button. If an individual did not agree to participate, the survey was automatically closed.

### 2.4 Data Analysis

SPSS 26.0 (IBM Corp., Armonk, NY, USA) was used for data analysis. Descriptive statistics were utilised to analyse the participant characteristics and the SRSS, DASS‐21, PSSS and BRS scores. Quantitative variables were presented as means (standard deviations [SDs]) and categorical variables as frequencies. A *t*‐test, one‐way analysis of variance and the chi‐squared test were used to compare groups. Pearson correlation coefficients were calculated to identify the relationship between the SRSS score and the DASS‐21, PSSS and BRS scores. A multiple linear stepwise regression analysis was conducted to determine the factors affecting sleep quality. The Mann–Whitney *U*‐test was utilised to compare the degree of sleep disorders between groups. P‐values of less than 0.05 were considered statistically significant.

### 2.5 Ethical Considerations

This study was approved by the Ethics Committee of the First Affiliated Hospital of Zhejiang University School of Medicine (IIT20220551B).

## 3 Results

A total of 573 participants completed the questionnaire, of whom 310 were working in Grade III hospitals and 263 in community hospitals. The mean age of the participants was 33.09 (SD = 7.45) years. More than 90% were women and were infected with COVID‐19. The mean SRSS score of the participants was 24.55 (SD = 6.55); mean DASS‐21 score, 21.50 (SD = 19.45); mean depression subscale score, 6.12 (SD = 6.47); mean anxiety subscale score, 6.85 (SD = 6.65); mean stress subscale score, 8.53 (SD = 7.54); mean PSSS score, 61.98 (SD = 12.80); mean family support subscale score, 21.06 (SD = 4.90); mean friend support subscale score, 20.84 (SD = 4.57); mean other support subscale score, 20.08 (SD = 4.50); and mean BRS score, 19.67 (SD = 3.72). Hospital grade (*F* = −3.856, *p* < 0.001), age (*F* = 3.053, *p* = 0.028), address (*F* = 3.245, *p* = 0.001), professional title (*F* = 3.707, *p* = 0.025), clinical frontline (*F* = 3.772, *p* < 0.001), length of service (F = −2.090, *p* = 0.037), COVID‐19 infection (*t* = −3.371, *p* = 0.010), and comorbidities (*t* = −4.37, *p* < 0.001) were found to influence the SRSS score. Conversely, sex(*t* = 0.534, *p* = 0.593), BMI (*F* = 0.204, *p* = 0.894), educational level (*t* = −1.623, *p* = 0.105), marital status (*t* = −1.284, *p* = 0.200), children (*t* = −1.697, *p* = 0.090), income (*F* = 1.019, *p* = 0.397), smoking status (*t* = −1.135, *p* = 0.257), drinking status (*t* = −1.325, *p* = 0.186), and religious beliefs (*t* = 0.939, *p* = 0.348) did not significantly influence the SRSS score (Table 1).

**TABLE 1:** Demographic characteristics of the SRSS and scores on each scale.

Variable	*N* (%)	Mean (SD)	F/T	*p*
Hospital grade				
Community hospital	263 (45.9)	23.42 (6.207)	−3.856	< 0.001
Tertiary hospitals	310 (54.1)	25.51 (6.684)		
Gender				
Male	31 (5.4)	25.16 (7.537)	0.534	0.593
Female	542 (94.6)	24.51 (6.493)		
Age				
20~	211 (36.8)	23.92 (6.612)	3.053	0.028
30~	238 (41.5)	24.67 (6.303)		
40~	105 (18.3)	24.84 (6.903)		
50+	19 (3.3)	28.47 (5.690)		
BMI				
< 18.5	68 (11.9)	24.94 (6.460)	0.204	0.894
18.5~	420 (73.3)	24.50 (6.533)		
24.0~	69 (12.0)	24.68 (6.559)		
≥ 28.0	16 (2.8)	23.63 (7.702)		
Education level				
College or below	108 (18.8)	23.63 (6.844)	−1.623	0.105
Bachelor degree or above	465 (81.2)	24.74 (6.466)		
Marriage				
Unmarried	166 (29.0)	24.00 (6.413)	−1.284	0.200
Married	407 (71.0)	24.77 (6.596)		
Children				
No	199 (34.7)	23.91 (6.254)	−1.697	0.090
Yes	374 (65.3)	24.89 (6.682)		
Place of residence				
City	456 (79.6)	24.95 (6.723)	3.245	0.001
Rural	117 (20.4)	22.99 (5.569)		
Professional title				
Entry level (Nurse + Nurse practitioner)	319 (55.7)	23.90 (6.279)	3.707	0.025
Medium level (Supervisor nurse)	227 (39.6)	25.30 (6.900)		
Advanced level (Associate Chief Nurse + Chief Nurse)	27 (4.7)	25.96 (5.913)		
Clinical front‐line				
No	251 (43.8)	23.39 (6.630)	3.772	< 0.001
Yes	322 (56.2)	25.45 (6.349)		
Length of service				
≤ 5年	191 (33.3)	23.74 (6.531)	−2.090	0.037
> 5年	382 (66.7)	24.95 (6.527)		
Covid‐19 infection				
No	55 (9.6)	21.75 (6.028)	−3.371	0.010
Yes	518 (90.4)	24.85 (6.535)		
Income				
< 2000	8 (1.4)	25.50 (7.464)	1.019	0.397
2000~	56 (9.8)	24.57 (6.564)		
4000~	142 (24.8)	23.72 (5.0.687)		
6000~	141 (24.6)	25.25 (6.834)		
> 8000	226 (39.4)	24.60 (6.828)		
Smoking				
No	569 (99.3)	24.52 (6.539)	−1.135	0.257
Yes	4 (0.7)	28.25 (7.762)		
Drinking				
No	542 (94.6)	24.46 (6.463)	−1.325	0.186
Yes	31 (5.4)	26.06 (7.853)		
Religious beliefs				
No	529 (92.3)	24.62 (6.587)	0.939	0.348
Yes	44 (7.7)	23.66 (6.046)		
Comorbidities				
No	525 (91.6)	24.19 (6.359)	−4.37	< 0.001
Yes	48 (8.4)	28.44 (7.351)		
Scores of each scale and dimension, mean (SD)				
SRSS (score)	24.55 (6.55)			
DASS‐21 (score)	21.50 (19.45)			
Depression (score)	6.12 (6.47)			
Anxiety (score)	6.85 (6.65)			
Stress (score)	8.53 (7.54)			
PSSS (score)	61.98 (12.80)			
Family support (score)	21.06 (4.90)			
Friend support (score)	20.84 (4.57)			
Others support (score)	20.08 (4.50)			
BRS (score)	19.67 (3.72)			

Abbreviations: BRS = Brief Resilience Scale, DASS‐21 = Depression, Anxiety and Stress Scale, PSSS = Perceived Social Support Scale, SD = Standard Deviation, SRSS = Self‐Rating Scale of Sleep.

Significant correlations were found between the SRSS score and the DASS‐21, PSSS and BRS scores (*p* < 0.01). The SRSS score was positively correlated with each DASS‐21 subscale score (*p* < 0.01) but negatively correlated with each PSSS subscale score (*p* < 0.01) and the BRS score (*p* < 0.01) (Table 2).

**TABLE 2:** Correlation between SRSS and DASS‐21, PSSS and BRS.

Questionnaires	Dimensions	SRSS	DASS‐21			PSSS		
			Depression	Anxiety	Stress	Family support	Friend support	Additional support
DASS‐21	Depression	0.464**	1					
	Anxiety	0.512**	0.796**	1				
	Stress	0.494**	0.850**	0.834**	1			
PSSS	Family support	−0.177**	−0.354**	−0.249**	−0.296**	1		
	Friend support	−0.185**	−0.363**	−0.281**	−0.330**	0.746**	1	
	Others support	−0.176**	−0.338**	−0.253**	−0.274**	0.744**	0.787**	1
BRS	Total	−0.433**	−0.538**	−0.505**	−0.558**	0.355**	0.411**	0.390**

Abbreviations: BRS = Brief Resilience Scale, DASS‐21 = Depression, Anxiety and Stress Scale, PSSS = Perceived Social Support Scale, SD = Standard Deviation, SRSS = Self‐Rating Scale of Sleep.

** *p* < 0.01.

The multiple linear hierarchical stepwise regression model was used to analyse the predictors of the SRSS score to reduce the interference of confounding factors. There was no significant evidence of multicollinearity noted in the model. The error range of the model was 0.286–0.982 (> 0.10), and the variance inflation factor was 1.008–3.375 (< 5). In Model 1, the following participant characteristics were added: hospital grade, age, address, professional title, clinical frontline, length of service, both COVID‐19 infection and comorbidities, comorbidities alone, clinical frontline, COVID‐19 infection, hospital grade and age; these factors independently predicted the SRSS score. The model explained 9.8% of the variance in the SRSS score (*F* = 13.430, *p* < 0.001). In Model 2, the DASS‐21 subscales related to the SRSS score were added; the explanatory power increased from 22.0% to 31.8% compared to the first stage (*F* = 39.090, *p* < 0.001). Anxiety and stress were found as independent predictors of the SRSS score. In Model 3, the PSSS subscales related to the SRSS score were added; consequently, the explanatory power increased by 0.5% from the previous stage, reaching 32.2% (*F* = 34.902, *p* < 0.001). Other support was observed as an independent predictor of the SRSS score. Finally, the BRS score was added to Model 4, increasing the explanatory power by 2.8% from the previous stage to 35.0% (*F* = 45.021, *p* < 0.001). There was no difference between stress and other support compared with the findings in Model 3. In this model, the BRS score was negatively correlated with the SRSS score, while anxiety, comorbidities, hospital grade, clinical frontline, age and COVID‐19 infection were positively correlated with the SRSS score (Table 3).

**TABLE 3:** Multiple hierarchical linear stepwise regression analysis of the SRSS of participants (*n* = 573).

Variables	Model 1					95% Cl	
	*B*	*SE*	*β*	*t*	*p*	Lower	Upper
*Model 1*							
Constant	16.54	1.31		12.67	0.000	13.98	19.11
Comorbidities	4.172	0.971	0.18	4.30	0.000	2.27	6.080
Clinical front‐line	2.43	0.55	0.18	4.39	0.000	1.34	3.51
Covid‐19 infection	3.02	0.89	0.14	3.41	0.001	1.28	4.76
Hospital grade	1.37	0.54	0.104	2.55	0.011	0.31	2.42
Ages	0.77	0.33	0.10	2.33	0.020	0.12	1.43
*Model 2*							
Constant	15.36	1.14		13.46	0.000	13.12	17.60
Anxiety	0.34	0.06	0.34	5.39	0.000	0.21	0.46
Comorbidities	2.84	0.85	0.12	3.32	0.001	1.16	4.51
Hospital grade	0.94	0.47	0.07	2.01	0.044	0.02	1.86
Stress	0.14	0.06	0.16	2.48	0.014	0.03	0.25
Clinical front‐line	1.563	0.49	0.12	3.22	0.001	0.611	2.52
Ages	0.83	0.29	0.10	2.86	0.004	0.26	1.40
Covid‐19 infection	1.75	0.78	0.08	2.25	0.025	0.22	3.28
*Model 3*							
Constant	17.33	1.50		11.57	0.000	14.384	20.27
Anxiety	0.33	0.06	0.34	5.30	0.000	0.21	0.453
Comorbidities	2.93	0.85	0.12	3.44	0.001	1.25	4.60
Hospital grade	1.06	0.47	0.08	2.25	0.025	0.14	1.99
Stress	0.12	0.06	0.14	2.18	0.030	0.01	0.23
Clinical front‐line	1.64	0.49	0.12	3.38	0.001	0.69	2.59
Ages	0.85	0.29	0.11	2.92	0.004	0.28	1.41
Covid‐19 infection	1.86	0.78	0.08	2.39	0.017	0.33	3.39
Others support	−0.11	0.053	−0.074	−2.02	0.044	−0.21	−0.00
*Model* 4							
Constant	23.89	1.85		12.94	0.000	20.26	27.51
Anxiety	0.350	0.04	0.36	8.91	0.000	0.27	0.43
BRS	−0.41	0.07	−0.23	−5.87	0.000	−0.54	−0.27
Comorbidities	2.76	0.83	0.12	3.32	0.001	1.13	4.39
Hospital grade	1.18	0.46	0.09	2.58	0.010	0.28	2.08
Clinical front‐line	1.56	0.47	0.12	3.30	0.001	0.63	2.49
Ages	0.81	0.283	0.10	2.88	0.004	0.26	1.37
Covid‐19 infection	1.98	0.76	0.09	2.63	0.009	0.500	3.47

*Note:* Hospital grade, Ages, Place of residence, Professional title, Clinical front‐line, Length of service, Covid‐19 infection, Comorbidities, included as control variables in the structural equation model. Model 1: *R* ^2^ = 0.106, adjusted *R* ^2^ = 0.098, *F* = 13.430, *p* < 0.001. Model 2: *R* ^2^ = 0.326, adjusted *R* ^2^ = 0.318, *F* = 39.090, *p* < 0.001. Model 3: *R* ^2^ = 0.331, adjusted *R* ^2^ = 0.322, *F* = 34.902, *p* < 0.001. Model 4: *R* ^2^ = 0.358, adjusted *R* ^2^ = 0.350, *F* = 45.021, *p* < 0.001.

Abbreviations: BRS = Brief Resilience Scale, CI = confidence interval, DASS‐21 = Depression, Anxiety and Stress Scale, PSSS = Perceived Social Support Scale, SRSS = Self‐Rating Scale of Sleep.

The participants older than 50 years were used as the reference group based on the results of Model 4. Further regression analysis revealed that this age group experienced significantly more sleep disorders than the other three age groups (Table 4).

**TABLE 4:** SRSS regression analysis in different age groups (*n* = 573).

Variables	Model					95% Cl	
	*B*	SE	*β*	*t*	*p*	Lower	Upper
Constant	28.474	1.494		19.058	0.000	25.539	31.408
20~	−4.554	1.560	−0.336	−2.920	0.004	−7.618	−1.490
30~	−3.806	1.553	−0.287	−2.451	0.015	−6.855	−0.756
40~	−3.636	1.624	−0.215	−2.239	0.026	−6.825	−0.447
50+	0						

*Note: R* ^2^ = 0.016, adjusted *R* ^2^ = 0.011, *F* = 3.053, *p* = 0.028.

Abbreviations: CI = confidence interval, SRSS = Self‐Rating Scale of Sleep.

The SRSS score of the participants ranged from 11 to 45. Comparing the SRSS items with a score greater than 3 points with the Chinese norm revealed significant differences in several areas: insufficient sleep time (*χ* ^2^ = 57.825, *p* < 0.001), poor sleep quality (*χ* ^2^ = 36.041, *p* < 0.001), difficulties in falling asleep (*χ* ^2^ = 47.018, *p* < 0.001), early awakening (*χ* ^2^ = 89.251, *p* < 0.001), medication (*χ* ^2^ = 15.148, *p* < 0.001) and psychophysiologic response after insomnia (*χ* ^2^ = 19.415, *p* < 0.001). The incidence of sleep disturbance (*χ* ^2^ = 49.47, *p* < 0.001) and degree of sleep disturbance (*Z* = −6.931, *p* < 0.001) were significant compared with the Chinese norm. Further analysis of the SRSS items with a score greater than 3 points among the nurses from community and tertiary hospitals showed significant differences in several areas: insufficient sleep time (*χ* ^2^ = 14.262, *p* < 0.001), difficulties in falling asleep (*χ* ^2^ = 8.319, *p* = 0.004), early awakening (*χ* ^2^ = 9.014, *p* = 0.003), medication (*χ* ^2^ = 15.120, *p* < 0.001) and psychophysiologic response after insomnia (*χ* ^2^ = 5.465, *p* = 0.019). There was a significant difference noted in the incidence of sleep disorders (*χ* ^2^ = 12.088, *p* < 0.001) but no significant difference in the degree of sleep disorders (*Z* = −1.093, *p* = 0.274) (Table 5).

**TABLE 5:** Comparison of sleep disorder status with Chinese norm and subgroups [N (%)].

Content	This study (*N* = 573)	Norm of norm (*N* = 13,273)	*χ* ^2^ */Z*	*p*	Community hospital (*N* = 263)	Tertiary hospitals (*N* = 310)	*χ* ^2^ */Z*	*p*
*SRSS item*								
Insufficient sleep time	190 (33.2)	2660 (20.1)	57.825	< 0.001	66 (25.1)	124 (40.0)	14.262	< 0.001
Poor sleep quality	83 (14.5)	1007 (7.6)	36.041	< 0.001	36 (13.7)	47 (15.2)	0.249	0.618
Daytime sleepiness	76 (13.3)	2064 (15.6)	2.198	0.138	27 (10.3)	49 (15.8)	3.796	0.051
Sleep hours	18 (3.1)	373 (2.8)	0.219	0.639	6 (2.3)	12 (3.9)	1.182	0.277
Difficulties in getting asleep	93 (16.2)	1075 (8.1)	47.018	< 0.001	30 (11.4)	63 (20.3)	8.319	0.004
Disrupted sleep	41 (7.2)	1214 (9.1)	2.642	0.104	17 (6.5)	24 (7.7)	0.350	0.554
Early awakening	126 (22.0)	1295 (9.8)	89.251	< 0.001	43 (16.3)	83 (26.8)	9.014	0.003
Dreaminess or nightmares/night terrors	63 (11.0)	1712 (12.9)	1.781	0.182	27 (10.3)	36 (11.6)	0.264	0.608
Medication	25 (4.4)	264 (2.0)	15.148	< 0.001	2 (0.8)	23 (7.4)	15.120	< 0.001
Psychophysiologic response after insomnia	228 (39.8)	4123 (31.1)	19.415	< 0.001	91 (34.6)	137 (44.2)	5.465	0.019
*Sleep disorders*								
No	226 (39.4)	7221 (54.4)	49.470	< 0.001	124 (47.1)	102 (32.9)	12.088	0.001
Yes	347 (60.6)	6052 (45.6)	49.470	< 0.001	139 (52.9)	208 (67.1)	12.088	0.001
*Degree of sleep disturbance*								
Mild	217 (37.9)	4725 (35.6)	−6.931	< 0.001	91 (34.6)	126 (40.6)	−1.093	0.274
Moderate	116 (20.2)	1270 (9.6)			45 (17.1)	71 (22.9)		
Severe	14 (2.4)	57 (0.4)			3 (1.1)	11 (3.5)		

Abbreviation: SRSS, Self‐Rating Scale of Sleep.

## 4 Discussion

In this study, we investigated the sleep status and characteristics of hospital nurses during the first explosive COVID‐19 outbreak in Zhejiang, China, after the relaxation of epidemic prevention and control measures. Sleep quality was measured using the SRSS, and its correlation with the DASS‐21, PSSS and BRS scores was evaluated. The SRSS items were also compared with the Chinese norm and within subgroups to identify severe sleep disorders among hospital nurses. The study revealed that the factors influencing nurses' sleep quality shifted following the change in epidemic policy. The mean SRSS score of participants in this study was 24.55 (SD = 6.55), indicating mild sleep issues on average. This score was slightly lower than the SRSS score of 25.13 (SD = 6.41) reported in Zhou et al. (2021)‘s study involving 1260 healthcare workers but significantly higher than the score of 21.00 (SD = 6.49) observed in Jiang et al. (2021)‘s study of 4245 healthcare workers. These variations highlight potential differences in sleep quality across different samples and time periods within the healthcare workforce.

First, we found that comorbidities, hospital grade, clinical frontline, age and COVID‐19 infection were independent predictors of the SRSS score in this study. Although the Omicron strain became the dominant strain of COVID‐19, significantly reducing the severity and mortality rates, the participants with comorbidities reported worse sleep quality (Al Maqbali 2021). This deterioration may be related to their concerns that comorbidities could worsen with COVID‐19 infection, hinder recovery or progress to severe disease. Such worries drove screen time searching for ways to cope or decompress (Elhai et al. 2020; Mishra et al. 2023). Additionally, negative media reports exacerbated their concerns, creating a vicious cycle (Lu et al. 2023). The urgent need for effective drugs, which were often out of stock, further increased their psychological burden and negatively affected their sleep.

The study also revealed variations in sleep quality according to hospital grade. The nurses working in Grade III hospitals experienced poorer sleep than their counterparts. This may be associated with the severity of the disease (Dong et al. 2017), work tasks (Huang et al. 2022) and frequent night shifts (Dong et al. 2017; Feng et al. 2021; Jin, Zhou, and Yuan 2022; Peng et al. 2022). Due to limited medical resources, community hospitals often refused to accept some critically ill patients with COVID‐19 and referred them to Grade III hospitals, which were required to admit all such patients. Therefore, the nurses in Grade III hospitals faced higher work pressures and physical demands. With the growing shortage of professional nursing staff for patients with severe COVID‐19, some nurses who had previously worked in intensive care units were reassigned to such units. Some nurses working in outpatient departments, supply rooms and physical examination centres were assigned to fever clinics, infusion rooms and general wards. These sudden changes in the working environment and workflow and the increasing difficulty of nursing care contributed to their stress and insomnia (Kalmbach, Anderson, and Drake 2018).

In this study, the nurses working on the COVID‐19 frontline reported worse sleep quality, consistent with several reports in the literature (Cleper et al. 2022; Jiang et al. 2021; Liu et al. 2022; Tu, He, and Zhou 2020). During the epidemic in the past 3 years, many Chinese nurses gained extensive experience in COVID‐19 prevention and control but lacked experience in treatment and direct nursing care. For most Chinese nurses, it was their first time to provide care to patients with COVID‐19. Many older adults with chronic diseases died suddenly after COVID‐19 infection, which further increased the psychological burden on the nurses. Studies have shown that older people have worse sleep quality, more severe insomnia symptoms and shorter sleep duration (Jin, Zhou, and Yuan 2022; Madrid‐Valero et al. 2017), consistent with our findings and with typical sleep changes that occur across the lifespan (Li, Vitiello, and Gooneratne 2022).

More than 90% of the nurses in this study were infected with COVID‐19, but the actual prevalence may be much higher because the government has stopped advocating COVID‐19 nucleic acid testing, and some asymptomatic or mild cases may have been missed. COVID‐19 infection has been shown to affect sleep, with sleep disturbance rates reaching 52%–75% (Jahrami et al. 2021, 2022). Reduced absolute lymphocyte count and increased neutrophil‐to‐lymphocyte ratio after COVID‐19 infection have also been found to be associated with increased risks of sleep dysfunction in patients with COVID‐19 (Salles and Mascarenhas Barbosa 2020). Additionally, COVID‐19 infection causes an inflammatory storm, leading to the release of various cytokines and chemokines in the blood, such as interleukins and TNFs. These immune modulators can induce circadian rhythm changes when they enter the brain or spinal cord (Shaik et al. 2023).

During the explosive national COVID‐19 outbreak, hospitals could not receive external assistance. Therefore, all available nurses had to continue working, except during the high‐fever period of COVID‐19 infection. This added stress further exacerbated sleep disorders. In addition, some studies have found that professional title (Huang et al. 2022; Zhou et al. 2022) and length of service (Al Maqbali 2021; Huang et al. 2022; Zheng et al. 2021) influences sleep quality, consistent with our findings. However, in Model 1, these factors, along with place of residence, were not significant. This may reflect some internal associations within the nurse characteristics in our study, such as nurses with high professional titles being relatively older and having been in the service for a long time, and the tendency for tertiary hospitals to be located in urban areas and community hospitals in rural areas.

Second, our study found that the DASS‐21 and PSSS scores differ from past findings, while the BRS score is generally consistent with past reports. Multiple studies have identified depression and anxiety (Al Maqbali 2021; Jiang et al. 2021; Lu et al. 2024; Sampaio, Sequeira, and Teixeira 2021; Tu, He, and Zhou 2020) as well as stress (Al Maqbali 2021; Lu et al. 2024; Sampaio, Sequeira, and Teixeira 2021) as factors influencing sleep disorders, consistent with our findings. However, only anxiety and stress were found to be significant predictors in our Models 2 and 3. In Model 4, only anxiety was noted as a predictor. Such results are consistent with those of a Brazilian longitudinal analysis of the impact of mental health on sleep patterns of healthcare professionals during the COVID‐19 pandemic (Dos Santos Alves Maria et al. 2023) and those of the study by Duan et al. (2022). According to Alvaro, Roberts, and Harris (2013), anxious individuals often experience sleep disruption and frequent awakening. This raises the question of why depression and stress did not appear as significant predictors in our model. The high prevalence of COVID‐19 infection among the nurses may have contributed to this outcome. Psychologically, the nurses appeared to have adapted to their challenging circumstances, demonstrating resilience and a pragmatic acceptance of the situation. This mindset was encapsulated by the sentiment: ‘Whether you get infected at work or in daily life, it is unavoidable.’ Such acceptance likely contributed to their ability to endure the situation without outwardly expressing significant complaints. Additionally, the relaxation of policies may have alleviated some of their pressures, such as reduced restrictions, fewer fears of criticism, no daily nucleic acid testing and simpler workflows. Consequently, their remaining anxiety may stem from the discomfort of illness and the reality of working while sick.

Our study also found that the family, friend and other support subscale scores in the PSSS were significantly associated with the SRSS score, consistent with several reports demonstrating that the PSSS score is a protective factor of sleep quality (Haseli et al. 2023; Huang et al. 2022; Jiang et al. 2021; Xiao et al. 2020). However, in Model 3, only other support remained significant. Jiang et al. (2021) considered other support to be more important, while Huang et al. (2022) highlighted family support. This may be related to the varying levels of social distancing restrictions during these two studies (i.e., relative freedom and absolute lockdown, respectively). When we added the BRS score to Model 4, other support was no longer significant, and the BRS score attenuated the effect of such support on the SRSS score. During the explosive national COVID‐19 outbreak, as both their family and friends were infected with COVID‐19, the support available to the nurses diminished. The nurses who were infected often hoped for sick leave, but due to the overwhelming number of patients and severe staffing shortage, they had to continue working, rely on themselves to address difficulties and accept more work tasks while ill. These factors may have contributed to the gradual disappearance of each PSSS subscale in Models 3 and 4.

The present study showed that the BRS score was negatively correlated with the SRSS score, suggesting that resilience is a protective factor for sleep (Cao et al. 2024; Pachi et al. 2023). Few studies have evaluated the relationship between resilience and sleep quality among medical staff (Cao et al. 2024; Hwang and Lee 2023; Kaye‐Kauderer et al. 2022; Pachi et al. 2023). This suggests that improving psychological resilience is crucial for people experiencing sleep difficulties. Resilience is a dynamic process of active adaptation and recovery from adversity (Bui et al. 2023). Research involving healthcare workers during the pandemic has consistently highlighted resilience as a safeguard against various mental health conditions, so it is critical to recognise the importance of organisational, social, personal and psychological factors when developing interventions to enhance the mental health of nurses and other healthcare workers (Pachi et al. 2023). All of these elements play a key role in promoting resilience and, consequently, mental health (Pollock et al. 2020).

Currently, there is no strong evidence on the most effective interventions to support resilience among healthcare workers during the pandemic (McCallum 2022). During the COVID‐19 epidemic, some factors may have improved the psychological resilience of nurses. For example, patients and their families rarely complained and understood that medical staff were working while sick. They were also supportive of government subsidies for healthcare workers, often expressed gratitude and maintained harmonious relationships with doctors. These factors may have contributed to improved occupational well‐being, increased stress resistance and enhanced psychological resilience among the nurses. In addition, hospital administrators can issue some notices during the epidemic. For example, the immediate family members of medical staff are hospitalised to implement the ‘green channel’ to provide their families with symptomatic treatment of COVID‐19 drugs and improve their professional status. Psychological health courses on ‘emotion self‐management’ and ‘stress resilience improvement’ were organised for nurses every year.

The sleep quality of nurses in this study showed significant differences compared to previous studies across related factors (e.g., depression, stress, family support, friend support and other forms of support). These differences may be attributed to variations in the cultural and national contexts of nurses, as well as differences in epidemic prevention and control policies. This finding underscores that sleep conditions and their influencing factors are subject to ‘dynamic’ changes based on situational and contextual shifts.

During the 3‐year epidemic, many nurses had limited opportunities to reunite with their families, especially around the culturally significant Spring Festival, which symbolises family unity and renewal. Recognising its importance, the government relaxed restrictions before the holiday, allowing nurses to return home. This emotional reprieve alleviated stress, reinforced resilience and improved their ability to adapt to policy changes, demonstrating the critical role of cultural and emotional factors in mitigating challenges.

Finally, we found that the scores for multiple SRSS items were higher than the Chinese norm, especially for the nurses in tertiary hospitals. These items included insufficient sleep time, poor sleep quality, difficulties in falling asleep, early awakening, medication and psychophysiologic response after insomnia. Further analysis showed that, except for poor sleep quality, the tertiary hospital nurses had significantly higher scores than the community hospital nurses.

To address the identified issues, health managers should focus on improving the sleep quality of nurses by implementing strategies, such as revising scheduling patterns, providing sleep hygiene education and offering appropriate support. Moreover, the government and society have opportunities for further improvement during similar outbreaks. For instance, the Chinese government could consider phased liberalisation of epidemic prevention measures (e.g., opening a province for 10–15 days at a time) to minimise nationwide disruptions and reduce strain on healthcare workers. Medical staff should receive early training on the treatment and care of COVID‐19 to enhance preparedness. Additionally, creating and promoting home‐based self‐management videos for mild cases through major media channels could help prevent overtreatment. Pharmaceutical manufacturers should also increase their stock of essential COVID‐19 medicines, such as fever reducers and cough medicines. Providing nurses with 3‐day antipyretics for timely fever management after infection is also crucial. These measures can extend the psychological buffer period and improve overall preparedness, helping to avoid panic buying and personal hoarding of medications.

## 5 Limitations

This study has several limitations. First, while the use of anonymous self‐assessment methods and internet‐based questionnaires encouraged participants to share their true feelings, the lack of face‐to‐face interviews or objective measures such as polysomnography resulted in subjective data, potentially affecting the reliability of the findings. Additionally, the use of convenience sampling due to time constraints led to a relatively small sample size, which may introduce bias. Future research should recruit larger, more representative samples to enhance the validity and generalisability of the results. Second, this study employed a cross‐sectional design, making it difficult to establish causal relationships between variables or to track changes in sleep status over time. Longitudinal studies, combined with follow‐up assessments such as telephone interviews, could provide a more comprehensive understanding of trends in sleep quality, particularly after the conclusion of the epidemic. Third, the data were limited to Zhejiang Province, China, which restricts the generalisability of the findings to the broader Chinese population. Furthermore, the study was conducted before the Spring Festival, a culturally significant period in China, further limiting its applicability to global populations. Fourth, the possibility of self‐reported sleep problems stemming from psychological distress or misperceptions linked to personality traits cannot be entirely ruled out (Fernandez‐Mendoza et al. 2011). Finally, the study did not examine how perspectives on the Spring Festival or the experience of working while sickness influenced sleep quality. Future research should explore additional dimensions, such as ‘emotional intelligence and self‐efficacy’ (Sarani et al. 2020) and ‘moral principles of religion’ (Mohammadi et al. 2021), to identify broader factors affecting sleep quality and to develop more effective strategies for improvement.

## 6 Conclusion

Anxiety, the BRS score, comorbidities, hospital grade, clinical frontline, age and COVID‐19 infection were found to be the independent predictors of sleep quality after the removal of control measures in Zhejiang Province, China. More than 90% of the nurses were infected with COVID‐19 for the first time within a short period and took on additional work tasks during illness, which made their SRSS scores higher than the Chinese norm. Thus, nurses in tertiary hospitals, in particular, require attention and support from hospital management. It is important to address their anxiety and enhance their psychological resilience. Both the government and society need to improve their response to similar future epidemics by offering extended psychological support and ensuring better psychological preparation. In addition, it is crucial to consider that changes in cultural background and epidemic status can influence nurse characteristics and other factors affecting sleep quality.

## Author Contributions

All authors have made substantial contributions to the manuscript to meet the criteria for authorship and have reviewed and agreed to the final version. Lifen Lu, Xiulan Shen and Gui Zheng conducted the study and the design, led the statistical analysis and drafting of the manuscript, revised it for critically important intellectual content and approved the final version for submission. Di Sheng, Yaling Zhu and Xiaowei Xia facilitated data collection, literature and statistics, and each made substantial contributions to drafting the manuscript and revising it for critically important intellectual content and approving the final version. Guanghui Chen and Jiali Liang conducted the data collection and approved the final version for submission.

## Ethics Statement

The First Affiliated Hospital of Zhejiang University School of Medicine's Ethics Committee gave its approval for this project (IIT20220551B).

## Conflicts of Interest

The authors declare no conflicts of interest.

## Data Availability

The data that support the findings of this study are available from the corresponding author upon reasonable request.
